# Superoxide dismutase 3 prevents early stage diabetic retinopathy in streptozotocin-induced diabetic rat model

**DOI:** 10.1371/journal.pone.0262396

**Published:** 2022-01-11

**Authors:** Ji-Yeon Lee, Mirinae Kim, Su Bin Oh, Hae-Young Kim, Chongtae Kim, Tae-Yoon Kim, Young-Hoon Park

**Affiliations:** 1 Catholic Institute for Visual Science, College of Medicine, The Catholic University of Korea, Seoul, Korea; 2 Department of Ophthalmology and Visual Science, Seoul St. Mary’s Hospital, College of Medicine, The Catholic University of Korea, Seoul, Republic of Korea; 3 Department of Dermatology, College of Medicine, The Catholic University of Korea, Seoul, Republic of Korea; Max Delbruck Centrum fur Molekulare Medizin Berlin Buch, GERMANY

## Abstract

**Purpose:**

To identify the effects of superoxide dismutase (SOD)3 on diabetes mellitus (DM)-induced retinal changes in a diabetic rat model.

**Methods:**

Diabetic models were established by a single intraperitoneal injection of streptozotocin (STZ) in Sprague-Dawley rats. After purification of the recombinant SOD3, intravitreal injection of SOD3 was performed at the time of STZ injection, and 1 and 2 weeks following STZ injection. Scotopic and photopic electroretinography (ERG) were recorded. Immunofluorescence staining with ɑ-smooth muscle actin (SMA), glial fibrillary acidic protein (GFAP), pigment epithelium-derived factor (PEDF), Flt1, recoverin, parvalbumin, extracellular superoxide dismutase (SOD3), 8-Hydroxy-2’deoxyguanosine (8-OHdG) and tumor necrosis factor-ɑ (TNF-ɑ) were evaluated.

**Results:**

In the scotopic ERG, the diabetic group showed reduced a- and b-wave amplitudes compared with the control group. In the photopic ERG, b-wave amplitude showed significant (*p* < 0.0005) reduction at 8 weeks following DM induction. However, the trend of a- and b-wave reduction was not evident in the SOD3 treated group. GFAP, Flt1, 8-OHdG and TNF-ɑ immunoreactivity were increased, and ɑ-SMA, PEDF and SOD3 immunoreactivity were decreased in the diabetic retina. The immunoreactivity of these markers was partially recovered in the SOD3 treated group. Parvalbumin expression was not decreased in the SOD3 treated group. In the diabetic retinas, the immunoreactivity of recoverin was weakly detected in both of the inner nuclear layer and inner plexiform layer compared to the control group but not in the SOD3 treated group.

**Conclusions:**

SOD3 treatment attenuated the loss of a/b-wave amplitudes in the diabetic rats, which was consistent with the immunohistochemical evaluation. We also suggest that in rod-dominant rodents, the use of blue on green photopic negative response (PhNR) is effective in measuring the inner retinal function in animal models of diabetic retinopathy. SOD3 treatment ameliorated the retinal Müller cell activation in diabetic rats and pericyte dysfunction. These results suggested that SOD3 exerted protective effects on the development of diabetic retinopathy.

## Introduction

Diabetic retinopathy (DR) is a major complication of diabetes, and remains the leading cause of preventable blindness in working-aged people [1]. DR is characterized by progressive changes in the retinal and choroidal microvasculature. In diabetes, chronically elevated levels of glucose drive inflammation and reactive oxygen species (ROS) production and cellular oxidative stress. Previous studies have identified that oxidative stress plays an important role in the development of DR [2, 3]. In diabetic patients, hyper- or hypoglycemia-induced ROS cause metabolic abnormalities including advanced glycation end-product formation, protein kinase C activation, and polyol pathway activation [3, 4]. If ROS are not properly neutralized they trigger activation the central inflammatory mediator nuclear factor-κB (NF-κB), amplifying inflammatory responses. This leads to the vascular endothelial growth factor (VEGF) secretion in the retina and increases the retinal vascular permeability.

The ROS production is mitigated by various antioxidant enzymes such as superoxide dismutase (SOD), glutathione reductase, glutathione peroxidase, and catalase [5]. Among these, SOD enzymes protect cells from the damaging effects of ROS by neutralizing the oxide radical (O_2_^-^) to molecular oxygen (O_2_) and H_2_O_2_ [6]. Three types of SOD isozymes are present in mammals: copper and zinc-containing SOD (Cu, Zn-SOD or SOD1), manganese-containing SOD (Mn-SOD or SOD2), and extracellular-SOD (EC-SOD or SOD3). Among them, extracellular SOD3 plays a central role in ROS regulation [6–8]. In the eye, anti-oxidative enzymes, including SOD, catalase, and glutathione peroxidase, exist in normal vitreous and choroid-retinal pigment epithelium complexes [9, 10].

Previous studies have proposed that antioxidant scavengers, including SODs, might be viable therapeutic targets for DR [2, 11–14]. Decreased SOD activity is associated with an increased risk of metabolic syndrome including diabetes [15]. Kanwar M *et al*. reported that SOD overexpression prevented diabetes-induced mitochondrial electron transport dysfunction and capillary degeneration [2]. Dietary supplementation with antioxidants can also alleviate diabetes-induced metabolic abnormalities [12–14]. Accordingly, the maintenance of SOD3 at high levels may alleviate oxidative stress in the retina and prevent DR. However, the efficacy and safety of SOD3 in DR have not been fully investigated.

This study aimed to evaluate the efficacy of SOD3 delivery through an intravitreal route in a rat model of STZ-induced diabetic retinopathy. In order to elucidate whether SOD3 exerts protective effect in DR, the histological and ERG findings were evaluated in an animal model of DR with intravitreal injection of recombinant SOD3.

## Materials and methods

### Animals

Male Sprague-Dawley rats (8 weeks old; weighing 250–300 g) were used in this study (Orient Bio Co., Seongnam-si, Gyeonggi-do, Korea). Animals were kept in a plastic cage in a climate-controlled laboratory with a 12 h-light/dark cycle.

All procedures performed in studies involving animals were in accordance with the ethical standards of the Institutional Animal Care and Use Committee (IACUC) and Department of Laboratory Animals (DOLA) in Catholic University of Korea at which the studies were conducted. Catholic University of Korea Songeui Campus accredited the Korea Excellence Animal laboratory Facility from Korea Food and Drug Administration in 2017 and acquired Association for Assessment and Accreditation of Laboratory Animal Care (AAALAC) International full accreditation in 2018.

All the animal procedures were carried out in accordance with the Laboratory Animals Welfare Act, the Guide for the Care and Use of Laboratory Animals and the Guidelines and Policies for Rodent experiments provided by IACUC in the School of Medicine, The Catholic University of Korea. (Approval number: CUMS-2019-0199-06). This article does not contain any studies with human participants performed by any of the authors.

### Induction of diabetes

A model of diabetes mellitus (DM) was established by a single intraperitoneal injection of streptozotocin (STZ; Sigma-Aldrich; 60 mg/kg body weight) in 0.05 M HCl-sodium citrate buffer solution (pH 5.5). Before the intraperitoneal injection of streptozotocin, the animals were placed in a gas chamber containing 2% isoflurane in oxygen. When unconscious, the animals were removed from the chamber but kept under anesthesia with a mask (1.5% isoflurane in oxygen). STZ injection was defined as day one.

Serum glucose was measured from the tail vein using an automated Accu-Check glucometer (Roche Diagnostics Ltd., Indianapolis, IN) 3 days following diabetes induction. When serum glucose measured > 250 mg/dL on day 3, the development of DM was confirmed, and the rats were used for further experiments. Body weight and serum glucose levels were recorded every week after DM induction.

### Recombinant SOD3 preparation

Human SOD3 was purified and its activity was assessed as described in the reference paper [16]. SOD3 expression plasmid was transfected into HEK-293T cells (ATCC^®^ CRL-11268^™^) with Attractene (Qiagen, Hilden, Germany) based on the manufacturer’s instructions. Five days after transfection, culture media containing SOD3 were collected, filtrated, and loaded onto HiTrap Chelating HP column (GE Healthcare, UK). After loading, the column was washed with more than 50 column volumes of washing buffer, 50 mM NaPO4, 500 mM NaCl, and 30 mM imidazole. Then, SOD3 was eluted by the elution buffer containing 50mM NaPO4, 500mM NaCl, 500mM imidazole, followed by dialysis in phosphate-buffered saline (PBS) containing 50 μM Cu^2+^/Zn^2+^ ions. The concentration of purified SOD3 was determined based on a bovine serum albumin (BSA) standard curve with a protein assay dye (Bio-Rad). The recombinant SOD3 was verified by western blot with SOD3 antibody.

The enzymatic activity of SOD3 was measured by the Superoxide Dismutase Assay Kit-WST (Dojindo Molecular Technologies, Rockville, MD, USA) following the manufacturer’s instructions. Briefly, a 20 μl sample or PBS (bank control) was mixed with 200 μl of 200 μM WST working solution and 20 μl of enzyme working solution. The mixtures were incubated for 20 min for developing the signal, which was read at A_450_ using a micro-plate reader. The SOD activity was determined from the dilution factor exhibiting 50% inhibition (IC_50_).

### Intravitreal injections

Intravitreal injections of SOD3 were performed at the time of STZ injection (diabetes induction day 1), and 1 and 2 weeks after STZ injection. Before the intravitreal injection, rats were anesthetized with intraperitoneal injection of a mixture of tiletamine plus zolazepam (30 mg/kg, Zoletil; Virbac, France) and xylazine (10 mg/kg, Rompun; Bayer, Germany). Topical proparacaine (0.5%) and mydriatics were applied to the eye before intravitreal injection. All intravitreal injections were preformed through the superior temporal sclera at 1 mm from the limbus. A sterile 34-G needle (WPI, Sarasota, FL, Sub-Microliter injection syringe and Nanofil needle) was used to deliver 10 μL (1000 Unit) of recombinant SOD3 solution per eye. For normal and diabetic controls, the same volume (10 μL) of normal saline was injected.

### Electroretinography recording

Rats were dark-adapted overnight (about 16 hours) before full-field ERG recordings were performed. Under dim red light (λ > 600 nm), rats were anesthetized with intraperitoneal injection of zolazepam and xylazine and placed in a Ganzfeld dome laying on the stage to ensure a stable position for recording. After moistening with a hydroxypropyl methylcellulose gel, gold-ring contact-lens electrodes were placed on the eyes. Two reference electrodes and one ground electrode were placed subcutaneously in the ears and in the tail, respectively. Stimuli were brief white flashes delivered via a Ganzfeld stimulator (UTAS-3000; LKC Technologies, Gaithersburg, MD, USA). Signals were amplified and filtered through a digital band-pass filter ranging from 5 Hz to 1 kHz to yield a- and b-waves. Luminescence intensity was calibrated by the manufacturer and controlled by the computer system. Scotopic responses are the mechanisms that are specifically adapted for dim or night vision [17]. Rats were first tested for rod-mediated scotopic ERG under dark adaptation exposed to flash intensity of 0.99 cd·s/m^2^. Averaged responses were obtained from 5 stimulus trials that were separated by a 10s inter-stimulus interval. The amplitude of the a-wave was measured from the baseline to the negative peak, and b-wave was measured from the trough of the a-wave to the following response peak. Photopic ERG was recorded on a white rod-suppressing background (30 cd/m^2^) after light adaptation for 10 min. Light-adapted flash responses were recorded at 6.28 cd·s/m^2^ (4 dB). Each measurement was an average of 5 responses obtained within a 10s inter-stimulus interval. Amplitudes of photopic b-waves were measured from the baseline to the positive peak. Scotopic and photopic ERG responses were each obtained from both eyes simultaneously. Photopic negative response (PhNR) was recorded under stimulus conditions: a brief 4 dB blue flash (peak wavelength 450 nm) at an intensity of 10 cd/m^2^ against a green background (peak wavelength 560 nm) of 10 cd/m^2^ (photopic units) [18]. The stimuli were blue flashes presented via a Ganzfeld integrating sphere after 15 min of light adaptation on blue and green background for a long duration (200 ms). Each recording was an average of 10 sweeps with an inter-stimulus interval of 0.4 s. PhNR amplitude was measured from the baseline to the trough of the negative peak following the b-wave. The peak latency of the PhNR was observed as the elapsed time between the instant of the flash and the point of maximal descent following the b-waves. The amplitudes were normalized by setting the maximal value as 100.

### Tissue preparation

The eyeballs were enucleated under anesthesia with zolazepam and xylazine in aseptic manner. The posterior halves of the globes were immersed in 4% paraformaldehyde in 0.1 M phosphate buffer (PB), pH 7.4. Whole retinas were dissected and immersed in the same fixative for 2 h. After fixation, the retinas were immersed in 30% sucrose, refrigerated overnight, and then flash-frozen in liquid nitrogen and stored at -70°C for preservation.

### Immunofluorescence staining

Retinal pieces were trimmed out from the central portion of the superior quadrant and rinsed with 0.01 M PBS, pH 7.4. After thorough washing, the retinal pieces were embedded in 4% agar and cut into 40-μm-thick vertical sections. The retinal sections were collected in culture wells and processed for immunofluorescent microscopy. To block nonspecific binding sites, sections were treated with buffer B (1% BSA, 0.2% bovine gelatin, 0.05% saponin in 0.01 M PBS) for 3 h on ice. Sections were then incubated with antibodies against monoclonal mouse anti-extracellular superoxide dismutase (SOD3, Santa Cruz Biotechnology, Inc., Dallas, TX, USA; dilution 1:250), monoclonal mouse anti- alpha smooth muscle actin (ɑ-SMA, Invitrogen, Waltham, MA, USA; dilution 1:800), monoclonal mouse anti-glial fibrillary acidic protein (GFAP, Merck Millipore, Burlington, MA, USA; dilution 1:2000), monoclonal mouse anti-8-Hydroxy-2’deoxyguanosine (8-OHdG, abcam, Cambridge, United Kingdom; dilution 1:200), polyclonal rabbit anti-pigment epithelium-derived factor (PEDF, abcam; dilution 1:200), polyclonal rabbit anti-Flt1 (abcam; dilution 1:200), polyclonal rabbit anti-recoverin (Merck Millipore; dilution 1:3000), polyclonal rabbit anti-parvalbumin (Swant, Burgdorf, Switzerland; dilution 1:5000) and polyclonal rabbit tumor necrosis factor-alpha (TNF-α, Invitrogen; dilution 1:700) overnight at 4°C. After washing with 0.01M PBS, immunoreactivity was visualized using secondary antibodies including the species-appropriate Cy3-conjugated donkey anti-mouse and rabbit IgG (Jackson ImmunoResearch, West Grove, PA, USA; dilution 1:3000) for 2 h at room temperature. Before mounting, cell nuclei were counterstained with 4′,6-Diamidine-2′-phenylindole dihydrochloride (DAPI, Thermo Scientific, Waltham, MA, USA; dilution 1:1000). After washing in 0.1M phosphate buffer (PB), sections were mounted on a glass slide with mounting medium (Dako, Santa Clara, CA, USA).

### Confocal microscopy

Immunofluorescent staining was evaluated via confocal laser scanning microscopy (LSM 800 Meta, Carl Zeiss Co. Ltd., Germany). Fluorescent images were captured with red (red: excitation 650 nm, emission 647~700 nm) at 240× and PEDF images were also shown at 400× magnification power. Captured images were converted to JPEG.

### Quantitative and statistical analysis

Immuno-labeled profiles were evaluated by counting approximately 15 randomly selected areas (200 × 200 μm per field) of each of the stained tissue sections at 240× magnification using a color image analyzer (ZEN 3.1 program for Windows). GraphPad Prism v8.4.2 software (GraphPad Software Inc., San Diego, CA, USA) was used to record and analyze the data collected. Statistical analyses were used ANOVA and Student’s t-test. All data are presented as the mean ± standard error of the mean (SEM). The differences were considered statistically significant at *p* < 0.05.

## Results

### Body weight and serum glucose levels

**Fig 1:**
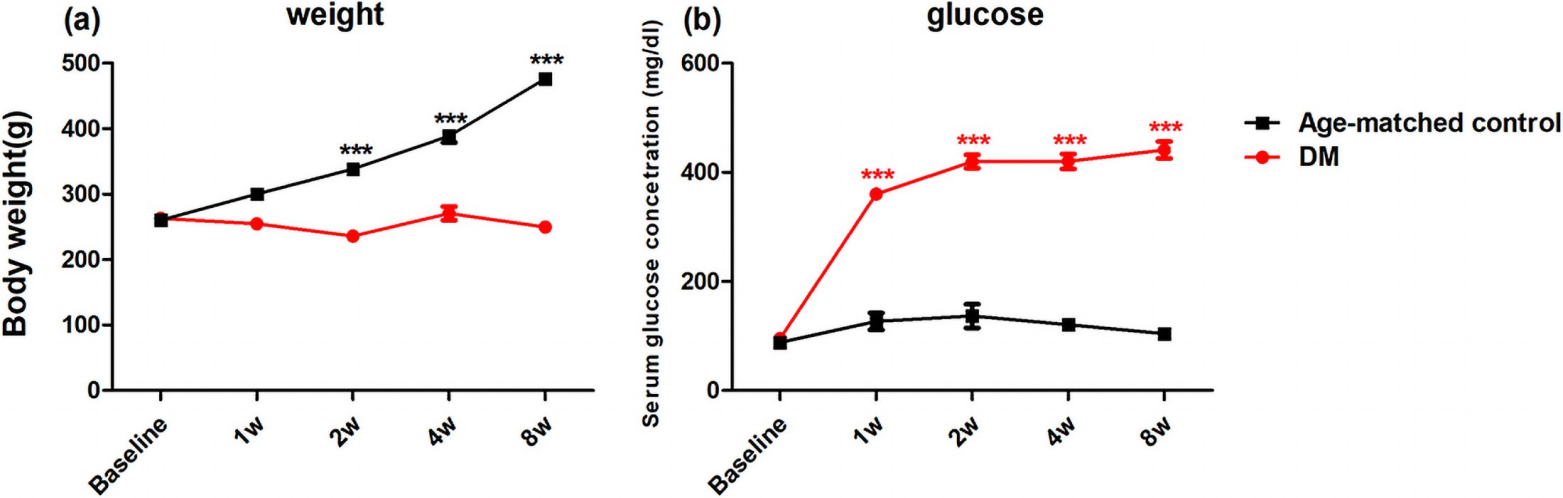
Body weight and serum glucose levels of age-matched control and diabetic rats over time. (a) Body weight and (b) serum glucose levels of diabetic rats compared to age-matched control rats up to 8 weeks after STZ injection. The mean body weight of rats in the diabetic group was lower than the age-matched control group (*p* < 0.0001). The serum glucose level was strikingly increased in the diabetes group compared with age-matched control group as early as the first week following STZ injection (*p* < 0.0001). Data are expressed as means ± SEM. n = 12 in the control group and DM group in each time point. *** *p* < 0.0001 versus age-matched control group.

The body weights and serum glucose levels of rat models were recorded to confirm the successful establishment of the diabetic rat models. The mean body weight of rats in the diabetic group was lower than the age-matched control group (*p* < 0.0001, Fig 1a). The serum glucose level was strikingly increased in the diabetes group compared with age-matched control group as early as the first week following STZ injection (*p* < 0.0001, Fig 1b).

### ERG of scotopic responses

**Fig 2:**
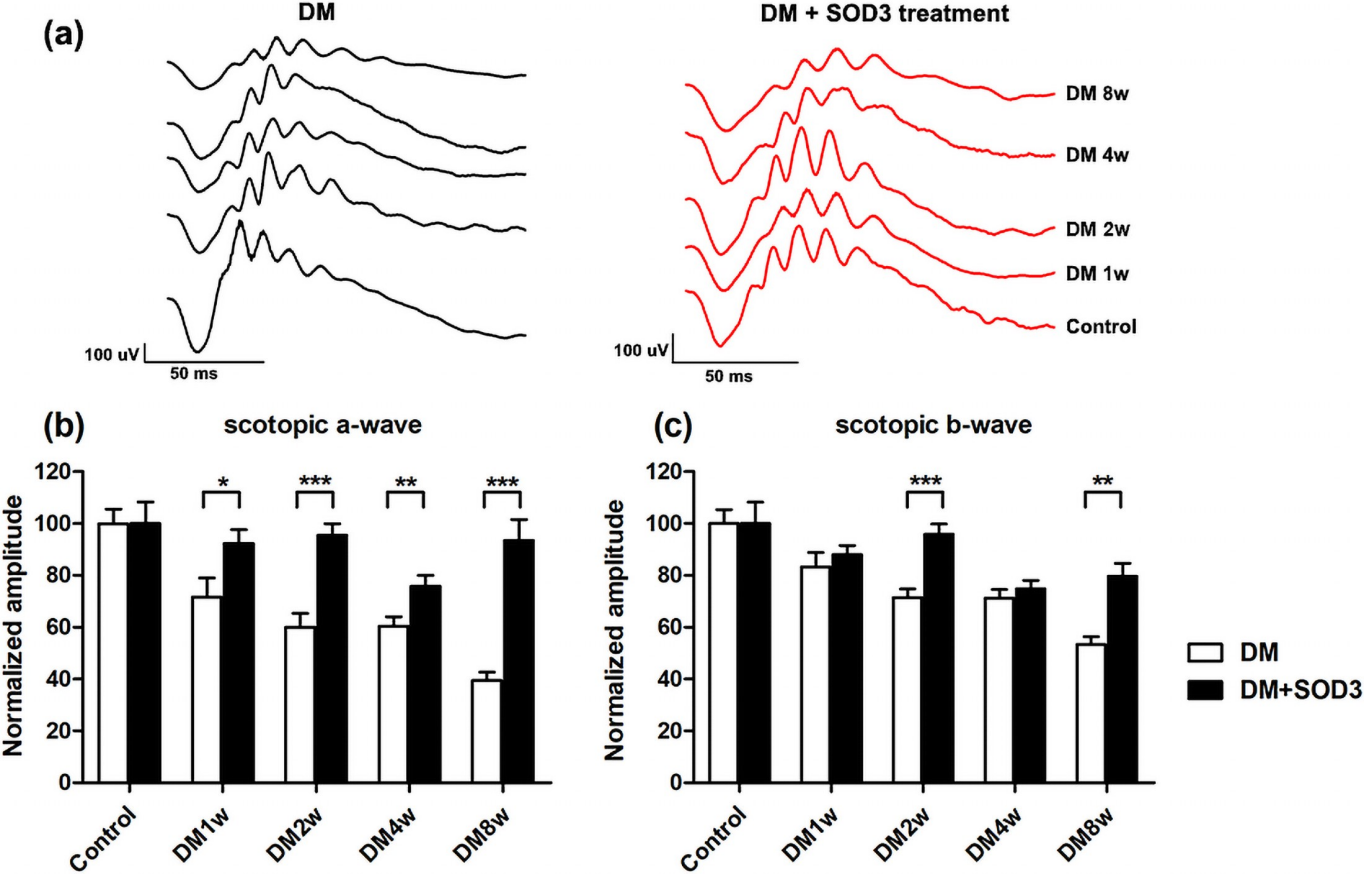
Measurement of scotopic electroretinography (ERG) amplitudes over time. STZ-induced DM rats were treated with or without SOD3. (a) Representative traces of scotopic ERG in the DM group and DM treated with SOD3 treatment group. Normalized measurement of (b) a-wave and (c) b-wave amplitudes by scotopic ERG. In scotopic ERG responses, the diabetic group showed significantly reduced a- and b-wave amplitudes compared with the DM + SOD3 injected group at all-time points. In normalized graphs, SOD3 administration has no effect on the normal retina in the control group. The diabetic group (white box) showed significantly reduced a- and b-wave amplitudes compared to the DM + SOD3 injected group at all-time points. However, the reduction of scotopic a- and b-waves were not distinct in the SOD3 injected group (black box). Measurements are expressed as the mean normalized amplitude ± SEM (n = 8 in the control group and in each time group). * *p* < 0.05, ** *p* < 0.002 and *** *p* < 0.0001.

Fig 2a shows the representative scotopic ERG traces. In the scotopic ERG responses, the diabetic group showed significantly reduced a-wave amplitudes compared to the diabetic + SOD3 injected group at all-time points (Fig 2b).

However, the reduction of scotopic a- and b-waves were not distinct in the diabetic group showed compared to the SOD3 injected group. Scotopic a- and b-wave amplitudes were not significantly reduced compared to the diabetic group at 1 week and 4 weeks after DM induction (all *p* > 0.05, Fig 2c). Only measurements at 2 and 8 weeks after DM induction were statistically reduced compared to the diabetic+ SOD3 injected group (*p < 0.05, **p < 0.002, ***p < 0.0001, Fig 2c).

### ERG of photopic responses

**Fig 3:**
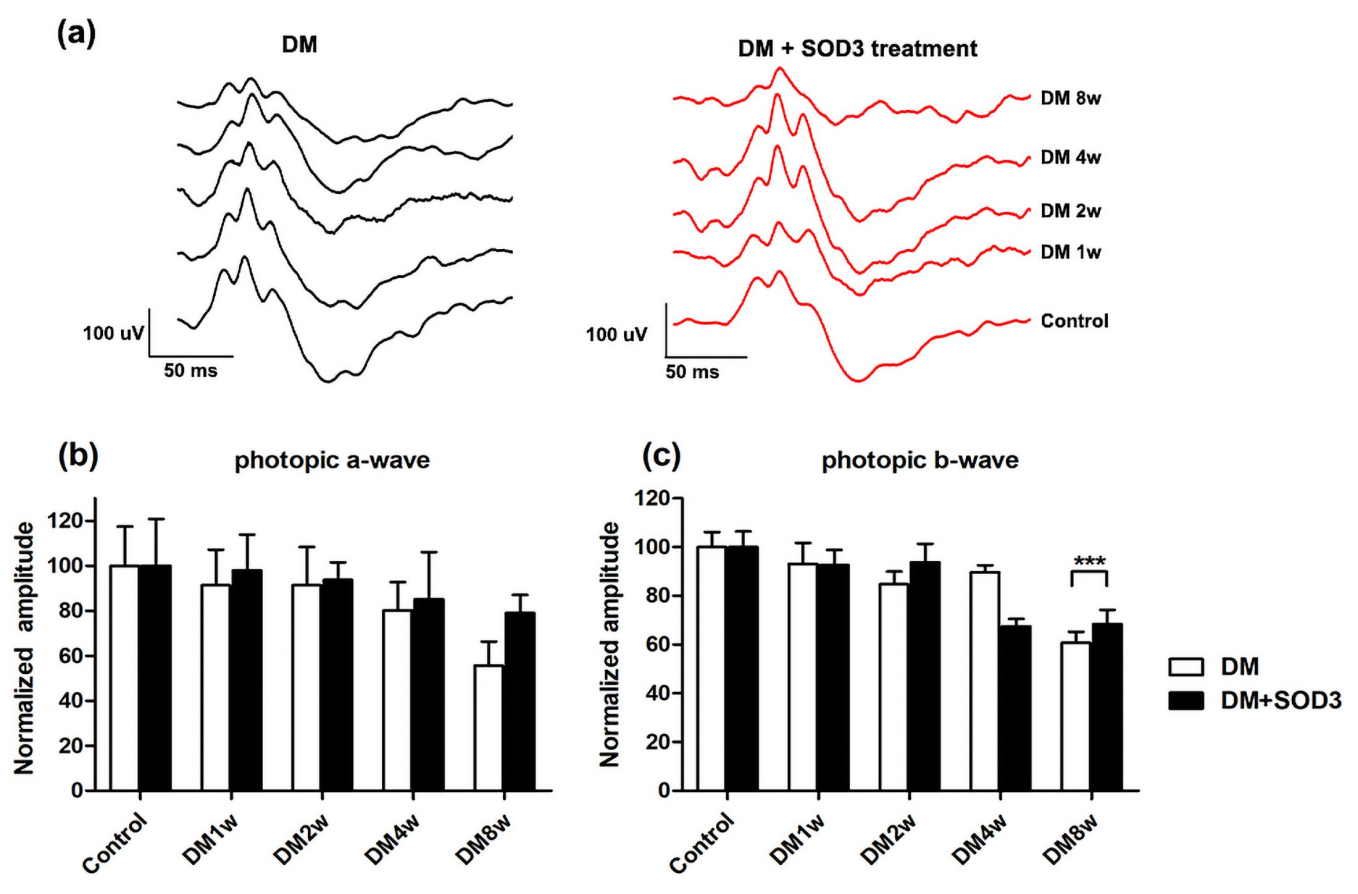
Changes in photopic electroretinography amplitudes over time. STZ-induced DM rats were treated with or without SOD3. (a) Representative traces of photopic ERG in DM group and DM treated with SOD3 treatment group. (b) a-wave amplitude and (c) b-wave amplitude was measured and normalized at different time points by photopic ERG. In photopic responses, we observed negligible a-wave amplitudes in both groups. The b-wave amplitude gradually reduced after DM induction, and the b-wave amplitude showed significant reduction at 8 weeks after DM induction (*p* < 0.0001). However, the trend of b-wave reduction was not evident in the SOD3 injected group. Measurements are expressed as the mean normalized amplitude ± SEM (n = 8 in the control group and in each time group). *** *p* < 0.0001.

Fig 3a shows representative photopic ERG traces. In the photopic responses, negligible a-wave amplitudes were observed in the diabetic and diabetic + SOD3 injected groups, and there were no statistical significances (all *p* > 0.05, Fig 3b).

The b-wave amplitude gradually reduced after DM induction, and the b-wave amplitude showed significant reduction at 8 weeks after DM induction (***p <0.0001, Fig 3c). The b-wave amplitudes were statistically reduced compared to the diabetic and diabetic + SOD3 injected groups at 8 weeks after DM induction (***p < 0.0001, Fig 3c).

### Photopic negative responses (PhNR)

**Fig 4:**
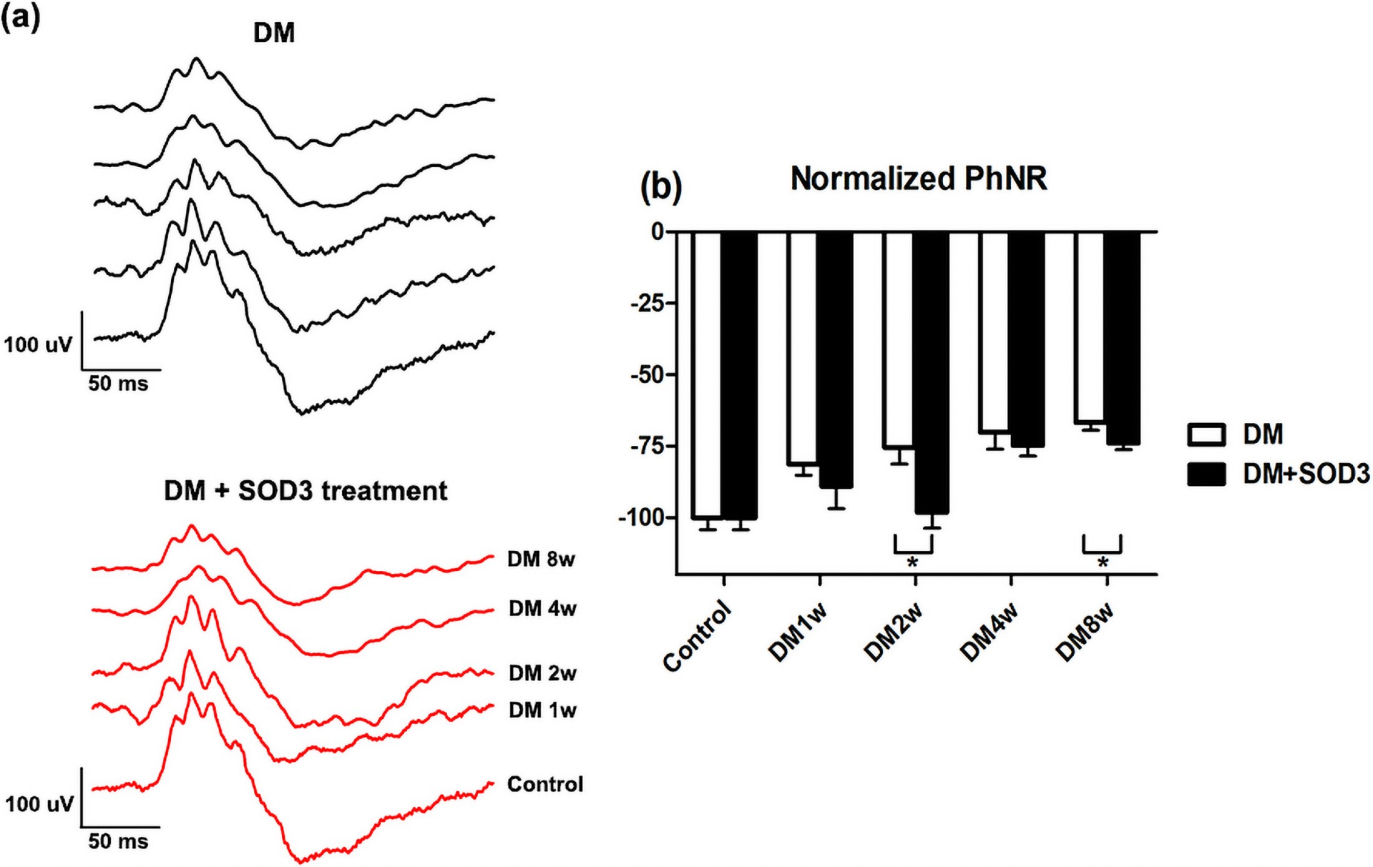
The changes in PhNR over time. PhNR was measured in STZ-induced DM rats treated with or without SOD3. (a) Representative traces of PhNR in DM group and DM treated with SOD3 treatment group. Normalized measurement of PhNR over time in (b) DM group and DM treated with SOD3 treatment group. In the DM group, the PhNRs amplitude showed significant reduction from week 1, and continued to decrease up to 8 weeks (all *p* < 0.05). Conversely, the SOD3 treatment group showed no reduction in PhNR amplitudes at early time points (week 1 and week 2) and was only significantly reduced at later time points (8 weeks after DM induction (*p* < 0.05)). Measurements are expressed as the mean normalized amplitude ± SEM (n = 8 in the control group and in each time group). * *p* < 0.05.

Fig 4a shows the representative traces of PhNR. In the PhNR amplitude, the diabetic group showed significantly reduced compared to the diabetic + SOD3 injected group at 2 weeks and 8 weeks (*p < 0.05, Fig 4b).

In the DM group, the PhNR amplitude showed significant reduction from week 1, and continued to decrease up to 8 weeks (white box, Fig 4b). Conversely, the SOD3 injected group showed no reduction in PhNR amplitudes at early time points (week 1 and week 2) and was only significantly reduced at later time points (4 and 8 weeks after DM induction (*p < 0.05, black box, Fig 4b)).

### Immunoreactivity

**Fig 5:**
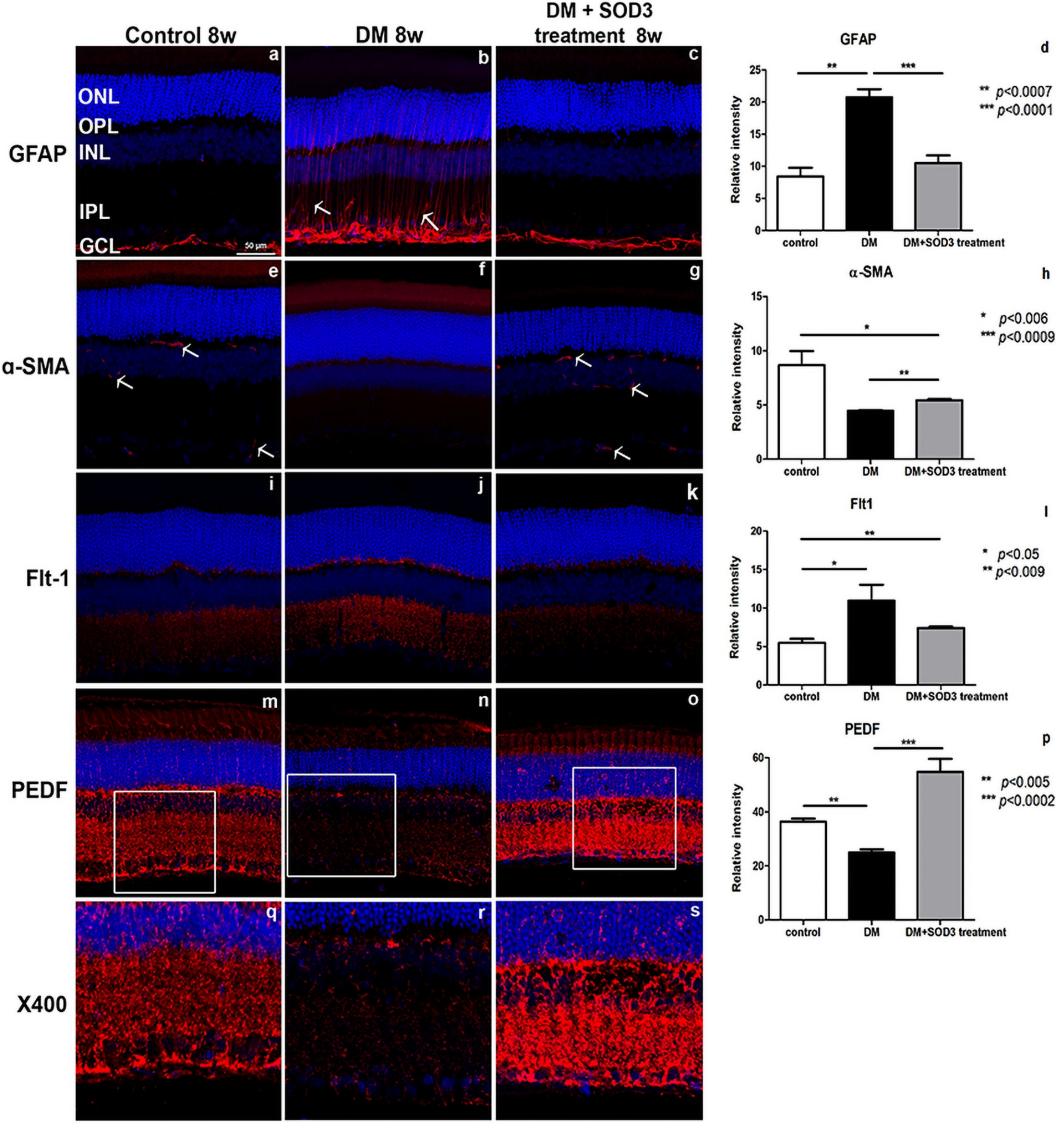
Immunofluorescence of rat retinas. The control (non-diabetic) retinas were compared to the retinas from STZ-induced DM rats treated with or without SOD3 by confocal microscopy. GFAP, α-SMA, Flt-1, PEDF (red), nuclei (DAPI, blue). Quantification of the relative intensity for GFAP (d), ɑ-SMA (h), Flt-1 (l) and PEDF (p) staining in the retinas. Measurements are expressed as the mean ± SEM (n = 8 in the control group and in each time group). GCL, ganglion cell layer; IPL, inner plexiform layer; INL, inner nuclear layer; ONL, outer nuclear layer; OPL, outer plexiform layer. Scale bar indicates 50 μm. * *p* < 0.05, ** *p* < 0.01, *** *p* < 0.001, **** *p* < 0.0005.

The astrocytes processes and the end feet of Müller glial cells, labeled by GFAP, were restricted to the retinal nerve fiber layer (RNFL) in the control group (Fig 5a). However, GFAP immunoreactivity was augmented and variably extended into the outer plexiform layer (OPL) in the diabetic retina (Fig 5b, white arrows, compared with 5a and 5c). In contrast, the diabetic retina treated with SOD3 had GFAP immunoreactivity restricted to the RNFL and ganglion cell layer (GCL) (Fig 5c), similar to the controls.

Pericytes express ɑ-SMA and are located in OPL, outer nuclear layer (ONL) and GCL in the control group (Fig 5e, white arrows). In the diabetic retinas, reduced ɑ-SMA immunoreactivity was observed suggesting loss of pericytes in DR (Fig 5f) [19], but ɑ-SMA was partially recovered in the SOD3 treated retinas (Fig 5g, white arrow, compared with 5f). In diabetic retinas, Vascular Endothelial Growth Factor receptor 1 (VEGFR1)/Flt1, which is normally found on vascular endothelial cells, showed higher immunoreactivity and a punctate expression pattern in the OPL and GCL. Flt1 was increased in the diabetic retinas (Fig 5j, compared with 5i and 5k).

PEDF immunoreactivity was localized to the RGC layer. PEDF expression was substantially decreased in the diabetic retinas, especially at DM 8 weeks (Fig 5n, compared with 5m) [20]. SOD3 treatment prevented the reduction in PEDF in diabetic rats (Fig 5o, * *p* < 0.05, ** *p* < 0.01, *** *p* < 0.001, **** *p* < 0.0005). Fig 5q, 5r and 5s images are shown at 400× magnification power.

**Fig 6:**
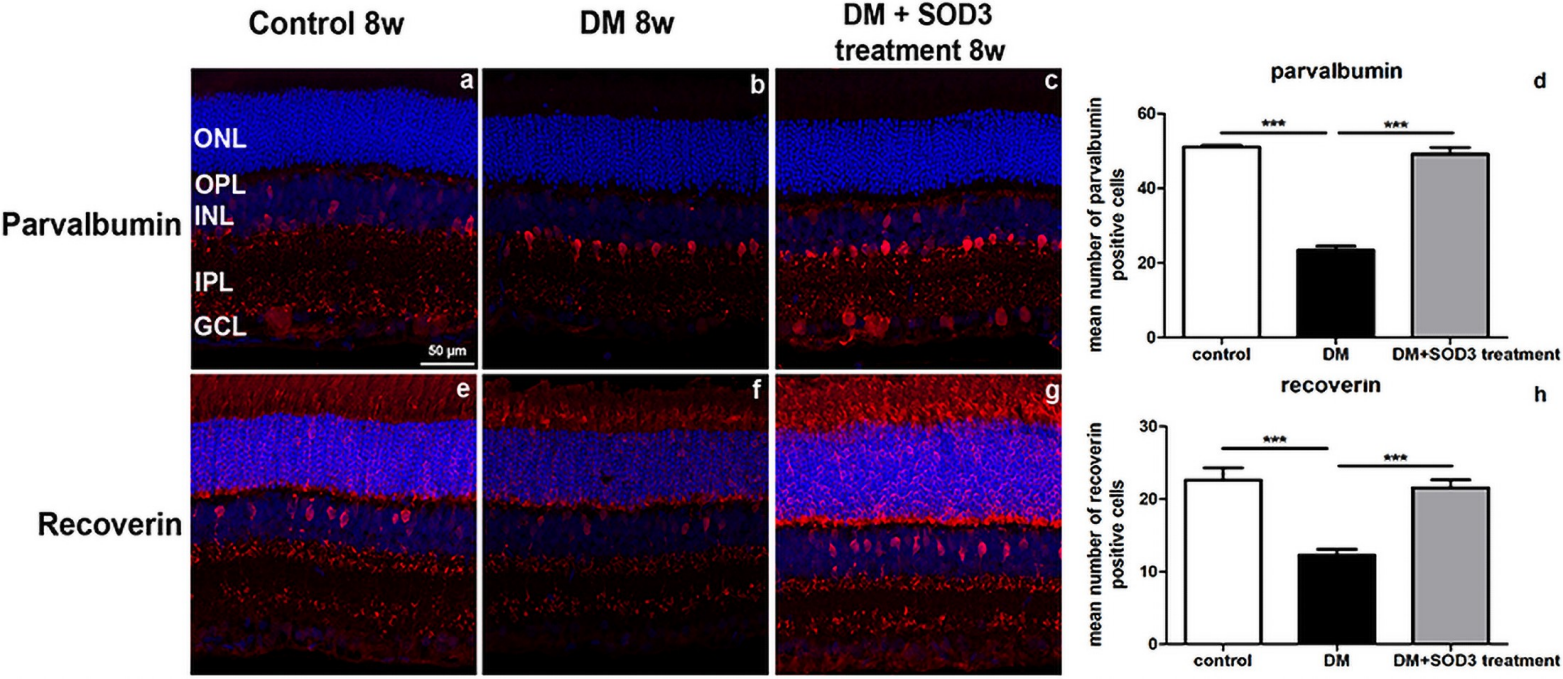
Confocal microscopic analysis of control rats and STZ-induced DM rats treated with or without SOD3. Immunofluorescence was used to visualize parvalbumin, recoverin (red) and nuclei (DAPI, blue). Quantification of the numbers for parvalbumin and recoverin positive cells staining in the retinas (Fig 6d and 6h). Measurements are expressed as the mean ± SEM (n = 8 in the control group and in each time group). GCL, ganglion cell layer; IPL, inner plexiform layer; INL, inner nuclear layer; ONL, outer nuclear layer; OPL, outer plexiform layer. Scale bar indicates 50 μm. *** *p* < 0.0001.

As previously reported [21], parvalbumin was localized to the AII amacrine cells within the INL, IPL and GCL in control retinas (Fig 6a). In the diabetic group, the expression was markedly reduced in lobular appendages of OFF lamina of the IPL and the dendrites near GCL (Fig 6b). Expression of parvalbumin was not decreased in diabetic rats treated with SOD3 (Fig 6c, compared with Fig 6b).

Recoverin is normally detected in cone bipolar cells with large somata at the outer border of INL and axon branch terminals in sublamina *a*, *b* in IPL (Fig 6e). In the diabetic retinas (Fig 6f), the expression of recoverin was weakly detected in both of INL and IPL compared with the control retina (Fig 6e) but not in the SOD3 treated group (Fig 6g, *** *p* < 0.0001).

**Fig 7:**
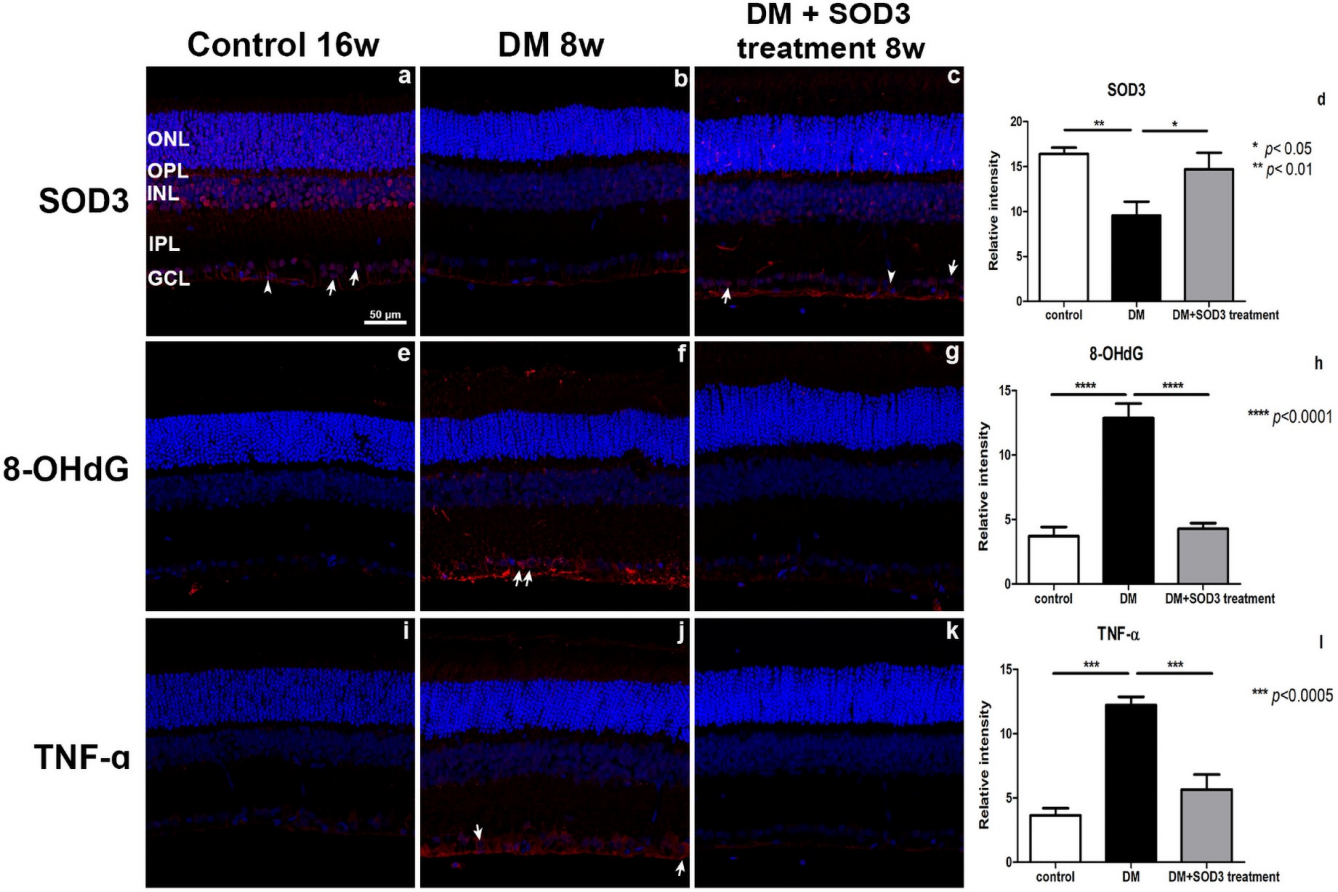
An evaluation of retinal SOD3, oxidative stress and inflammation imunofluorescence of rat retinas. The control (non-diabetic) retinas were compared to the retinas from STZ-induced DM rats treated with or without SOD3 by confocal microscopy. SOD3, 8-OHdG and TNF-ɑ (red), nuclei (DAPI, blue). Quantification of the relative intensity for SOD3 (d), 8-OHdG (h) and TNF-ɑ (l) staining in the retinas. Measurements are expressed as the means ± SEM (n = 8 in the control group and in each time group). GCL, ganglion cell layer; IPL, inner plexiform layer; INL, inner nuclear layer; ONL, outer nuclear layer; OPL, outer plexiform layer. Scale bar indicates 50 μm. * *p* < 0.05, ** *p* < 0.01, *** *p* < 0.0005, **** *p* < 0.0001.

SOD3 was showed ganglion cells (arrow) and a few glial cells (arrow head) in control retina (Fig 7a), but in the diabetic retinas (Fig 7b), the expression of SOD3 was clearly decreased compared with the control retinas. Expression of SOD3 was recovered in the diabetic rats treated with SOD3 (Fig 7c).

Oxidative stress marker 8-OHdG was expressed ganglion cells (arrow) and RNFL in the diabetic retinas (Fig 7f) but markedly decreased in the control retinas (Fig 7e) and diabetic rats treated with SOD3 retinas (Fig 7g).

TNF-ɑ positive cells were also detected strongly ganglion cells (arrow) in the diabetic retinas (Fig 7j) compared with the control retinas (Fig 7i). In the SOD3 treated retina group (Fig 7k) was decreased in the control retinas level (Fig 7, * *p* < 0.05, ** *p* < 0.01, *** *p* < 0.001, **** *p* < 0.0005).

## Discussion

This study identified a short-term effect of human SOD3 on diabetes-induced retinal changes in a diabetic rat model. In the present study, the SOD3 treatment attenuated the loss of a- and b- wave amplitudes on scotopic ERG and b-wave amplitudes on photopic ERG in diabetic rats, which was consistent with our immunohistochemical evaluation. SOD3 treatment also ameliorated retinal Müller cell activation in diabetic rats and pericyte dysfunction. These results suggested that SOD3 exerted a protective effect on the development of DR.

SOD3 is a highly conserved enzyme that is widespread in the human body and plays a fundamental role in protecting cells from oxidative stress [22]. Alterations in SOD3 activity and its expression have been observed in pathologic processes in various diseases including myocardial infarction, chronic kidney disease, type 2 DM, osteoarthritis, and malignancy [23–27]. In 2018, Zhao *et al*. [26] observed that reduction of serum SOD3 activity was associated with the progression of DR in 343 patients. Serum SOD3 activity has also been negatively correlated with severity of DR, HbA1c and duration of DM [26].

The role of oxidative stress in the pathophysiology of DR is well established [28–31]. Use of antioxidant enzymes such as SOD, catalase and glutathione peroxidase has previously been hypothesized as therapies to treat DR. SOD1 and SOD2 are intracellular enzymes that are localized in the cytoplasm and mitochondria [29]. SOD3 is a secreted extracellular enzyme which localizes to the interstitial matrix, mainly in blood vessels [30]. We hypothesized that the predominance of SOD3 in the vessel walls suggested that this SOD may have a more important role than the other isozymes in protecting against oxidative stress in vascular diseases including DR. Furthermore, SOD3 is known to have not only antioxidant properties but also anti-angiogenic, anti-proliferative, anti-chemotactic and anti-inflammatory properties, making it an attractive therapeutic target for DR [31, 32].

Pericyte loss is an early event in DR that occurs before vascular histopathology can be visualized [33, 34]. Our results clearly demonstrate that SOD3 ameliorates pericyte dysfunction and halts early capillary changes and inflammatory processes. The level of PEDF was attenuated in the SOD3-treated diabetic retinas. PEDF has neuroprotective and antiangiogenic functions in the mammalian eye, and was recently shown to also have anti-inflammatory properties [35].

Also, we found that in the diabetic retinas, the immunoreactivity of recoverin was weakly detected in both of the inner nuclear layer and inner plexiform layer compared to the control group but not in the SOD3 treated group. Recoverin is a neuronal calcium-binding protein that is primarily detected in the photoreceptor cells of the eye [36]. It plays a key role in the inhibition of rhodopsin kinase, a molecule which regulates the phosphorylation of rhodopsin [37]. A reduction in this inhibition helps regulate sensory adaptation in the retina, since the light-dependent channel closure in photoreceptors causes calcium levels to decrease, which relieves the inhibition of rhodopsin kinase by calcium-bound recoverin, leading to a more rapid inactivation of metarhodopsin II.

SOD activity is one of the most important antioxidant defense systems in retina. Retinal oxidative stress is one of the common secondary features of many retinal diseases including DR. Oxidative stress plays an important role in diabetic complications [38]. ROS can induce DNA damage. 8-OHdG is an oxidized derivative of deoxyguanosine and one of the major products of DNA oxidation [39]. 8-OHdG concentration within a cell represents intracellular ROS level. DR has regarded as a stimulator for the release of pro-inflammatory cytokine including such as TNF-ɑ and interleukin-1β (IL-1β). In this study, we studied 8-OHdG, which is one of the markers of oxidative stress, and TNF-ɑ, which is one of the markers of inflammation. We confirmed that treatment of SOD3 in STZ-induced DM suppressed oxidative stress and inflammation at an early stage, and suggested the potential for long-term effects in DM.

The STZ-induced diabetic rat model recapitulates only the non-proliferative stage of DR, limiting the applicability of our study. Although numerous diabetic animal models have shown significant retinal microvascular changes, none has DR that closely represent the specific features of human disease [40]. Previous studies in the STZ-induced animal model reported only early stage DR [41, 42], while one study detected the pre-proliferative stage of DR after 6 months [43]. Future studies will focus on addressing the long-term effect of SOD3 in the rat STZ-induced diabetes model.

In summary, this is the first experiment to apply the antioxidant SOD3 to the retina in DM, while combining the use of the blue on green PhNRs [18] reflecting inner retinal function changes in diabetic rats. Our study investigated the potential protective effect of SOD3 in DR. It should be mentioned that in rodent diabetic models, their usefulness is compared to that of scotopic ERG responses. Administering SOD3 to STZ-induced diabetic rats ameliorated oxidative stress-associated retinal damage. SOD3 treatment attenuated the loss of a- and b- wave amplitudes on ERG. SOD3 also ameliorated retinal Müller cell activation, pericyte dysfunction and neuroinflammation. Our study suggests that SOD3 protect against DR by modulating oxidative stress and inflammation. We also suggests that in rod-dominant rodents, the use of blue on green PhNRs is effective in measuring inner retinal function in animal models of diabetic retinopathy.
